# Anti-Tumor Activity of Expanded PBMC-Derived NK Cells by Feeder-Free Protocol in Ovarian Cancer

**DOI:** 10.3390/cancers13225866

**Published:** 2021-11-22

**Authors:** Minhua Chen, Yutong Li, Yu Wu, Siqi Xie, Jie Ma, Jingjing Yue, Rong Lv, Zhigang Tian, Fang Fang, Weihua Xiao

**Affiliations:** 1 The First Affiliated Hospital of USTC, Division of Life Sciences and Medicine, University of Science and Technology of China, Hefei 230001, China; chenmh@mail.ustc.edu.cn (M.C.); lyt1996@mail.ustc.edu.cn (Y.L.); wuyu@ustc.edu.cn (Y.W.); xiesq@mail.ustc.edu.cn (S.X.); mj223229@mail.ustc.edu.cn (J.M.); yuejingjing@mail.ustc.edu.cn (J.Y.); tzg@ustc.edu.cn (Z.T.); 2 Hefei National Laboratory for Physical Sciences at Microscale, CAS Key Laboratory of Innate Immunity and Chronic Disease, School of Life Sciences, University of Science and Technology of China, Hefei 230027, China; 3 Institute of Immunology, University of Science and Technology of China, Hefei 230027, China; 4 Engineering Technology Research Center of Biotechnology Drugs Anhui, University of Science and Technology of China, Hefei 230027, China; 5 Blood Transfusion Laboratory, Anhui Blood Center, Hefei 230031, China; lvrong612@126.com

**Keywords:** natural killer cell, ovarian cancer, ascites, cell immunotherapy, allogenic NK, immunotherapy

## Abstract

**Simple Summary:**

Natural killer (NK) cell-based cancer therapies have substantially reshaped modern strategies for clinical cancer treatment and have improved outcomes for countless patients, notably in hematologic malignancies. Unfortunately, its efficacy is limited against solid tumors due to poor infiltration and persistence in the tumor microenvironment. In this study, we show that peripheral blood mononuclear cell-derived NK cell cytotoxicity and persistence can be greatly enhanced to provide highly potent and durable anti-tumor activity through expansion in a feeder cell free-expansion system. Importantly, we show expanded-NK efficacy against three distinct forms of ovarian cancer in mouse models (i.e., solid tumors, abdominal metastatic tumors, and ascites).

**Abstract:**

Natural killer (NK) cells have shown great therapeutic potential against a wide range of cancers due to their pan-specific target recognition. Numerous reports indicate that NK cell immunotherapy is an effective therapeutic approach for treating hematological malignancies, but shows limited effects against solid tumors. In this study, several models of ovarian cancer (OC) were used to test the anti-cancer effects of NK cells derived from human peripheral blood mononuclear cells and expanded using a feeder cell-free expansion system (eNKs). The results show that eNKs exhibit potent inhibitory activity on tumor growth in different ovarian cancer xenograft mice (i.e., solid tumors, abdominal metastatic tumors, and ascites), importantly, in a dose-dependent manner. Moreover, adoptive transfer of eNKs resulted in significant reduction in ascites formation in OC peritoneal tumor models, and especially in reducing intraperitoneal ascites. We found that eNKs could migrate to the tumor site, retain their activity, and proliferate to maintain high cell counts in cutaneous xenograft mice. In addition, when increased the infusion with a high dose of 12 × 10^7^ cells/mouse, Graft-versus-host disease could be induced by eNK. These data show that eNK cell immunotherapy could be a promising treatment strategy for ovarian cancers, including solid tumors and ascites.

## 1. Introduction

Natural killer (NK) cells are effector cells of the innate immune system, which function in cytotoxic activity and cytokine production in anti-tumor responses [1]. However, native NKs in cancer patients are limited in number and show decreased functionality, so multiple immunotherapeutic approaches have been developed to overcome NK cell deficiency [2]. Among them, adoptive transfer cell therapy with allogeneic NK cells is considered to be one of the most effective methods for restoration of NK cells in patients. In patients, this approach can increase the number as well as the activity of NK cells, while avoiding inhibition due to self-histocompatibility antigens such as autologous NK cells [3,4]. An increasing number of clinical trials have revealed the effectiveness of adoptively transferred NK cells against hematologic malignancies [5,6]. However, the therapeutic effects of NK cells in treatment of solid malignancies have not been as effective as that for hematologic malignancies. Because of the relatively isolated physiological location and the specific immunosuppressive microenvironment of solid tumors, NK cells have difficulty in infiltrating into tumors, and moreover, their ability to proliferate and persist are disrupted once inside the tumor [7,8]. The resolution of these two obstacles would highly improve the therapeutic efficacy of NK cells against solid tumors. However, strategies using increased infusion doses of NK cells may increase NK cell efficacy, but may also raise the risk of T cell-related GvHD, especially when the cell products are mixed with the T cells [9,10]. Therefore, it remains unclear how high an infusion dose can be administered that is still safe and effective.

NK cell function is governed by a complex set of activating and inhibitory receptors that do not require tumor-specific antigens or antigen-presenting cell activation [11]. The expression of various ligands on cancer cells reportedly modulates the function of NK cells [12,13]. Therefore, screening and identification of cancer types that are sensitive to NK cell killing activity would improve the outcomes of strategic decision making for NK cell therapies. Studies have shown that human ovarian cancer cells express a high level of NK cell-activated receptor ligands (e.g., NKG2D, NCRs, and DNAM-1) and a low level of NK cell-inhibited receptor ligands (e.g., NKG2A/CD94, PD-1, and TIGIT), which suggest that NK cells may show high sensitivity to ovarian cancer cells [14,15].

Ovarian cancer (OC) is generally asymptomatic in the early stages, and as a result, 85% of patients are diagnosed in the advanced stages [14,16], with a 5-year survival rate of only 20% for OC patients with advanced stage III, and only 6% for OC patients with stage IV [17]. In addition, more than one-third of OC patients develop malignant ascites, which are associated with poor life quality and mortality [18]. Notably, ascites is also known to contribute to chemoresistance, metastasis, and decreased tumor resectability [19,20]. Unfortunately, there is still a lack of effective treatments for ovarian ascites. Approximately 60–80% of OC patients achieve complete remission after surgery and/or chemotherapy, whereas OC recurs in 60% of patients within 3 years and >50% of them show resistance to chemotherapy, with a 5-year survival rate of only 29% [21,22]. The emerging immunotherapies (such as cancer vaccines, immune checkpoint inhibitors, and adoptively transferred chimeric antigen receptor (CAR)-T cell) have brought hope for the development of new treatments for OC, but still have not been as successful as expected due to a lack of targetable antigens, the loss of human leukocyte antigen expression by tumors, and immunosuppression in the tumor or ascites microenvironments, etc. [15]. Nevertheless, NK cells can recognize (pan-specific tumor recognition) and kill tumors (via direct and indirect pathways) by different T cell-related mechanisms [5,23], thus suggesting that NK cells can serve as potential effectors against OC.

Several clinical trials have demonstrated that the number and functionality of NK cells in ascites or peripheral blood are positively correlated with the overall survival of patients [24]. However, NK cells are dysfunctional in most OC patients [25], and thus adoptively transferred NK cells are needed to improve NK function in OC patients. There are currently six clinical trials in which 31 patients have received adoptively transferred NK cells. Among these, 13% of the total 31 patients have shown at least partial response, 26% of the patients have reached stable disease, and 10% of patients report mild side effects [25,26]. In addition, no studies have examined whether NK cells can reduce ascites formation in OC patients. Although several preclinical studies have confirmed that adoptively transferred NK cells can reduce the OC burden in mice [14,27,28,29], the optimal safe dose for NK cell infusion and its efficacy against various forms of OC, such as solid ovarian tumors, ovarian abdominal metastases, and especially ovarian ascites, have not been reported.

In this study, we expanded and activated peripheral blood mononuclear cell (PBMC)-derived NK cells. We show that these expanded NKs (eNKs) can migrate to the tumor site and proliferate with no obvious inhibition by the immune system in an NCG mouse model of OC. We further evaluated the efficacy of eNKs in two representative cell line-derived xenograft models of OC. Our results thus demonstrated that adoptively transferred eNKs could inhibit tumor growth and reduce the ascites burden in vivo. Moreover, a high infusion dose of eNKs improved the therapeutic effects without inducing GvHD.

## 2. Materials and Methods

### 2.1. Cell Lines and Mice

A375 (RRID:CVCL_0132) and Skov3 (RRID:CVCL_0532) cell lines were bought from Zhigang Tian (University of Science and Technology of China), K562 (RRID:CVCL_0004), Molt4 (RRID:CVCL_0013) and Ho8910 (RRID:CVCL_6868) cell lines were purchased from the cell bank of the Chinese Academy of Sciences (Shanghai, China) and viably cryopreserved and stored in liquid nitrogen culture according to ATCC instruction. The base medium for K562 cell line is Iscove’s Modified Dulbecco’s Medium and supplemented with 10% FBS. The other cell lines were maintained in RPMI Medium Modified (HyClone, SH30809.01) supplemented with 10% FBS. The Skov3-luc and Ho8910-luc cell line were constructed by transfection of the luciferase-encoding vector into the Skov3 or Ho8910 cells, using the lentiviral packing system previously described [30], followed by stable cell selection. All the cell lines were identified with STR profiling by genewiz cpmpany (Suzhou, China).

Female NCG (NOD/ShiLtJGpt-Prkdc^em26Cd52^Il2rg^em26Cd22^/Gpt) mice were purchased from Jiangsu Gempharmatech. NCG mice were bred and kept in ultraclean barrier facilities, and used in accordance with the guidelines of experimental animals approved by the University of Science and Technology of China Animal Studies Committee.

### 2.2. NK Cell Expansion Ex Vivo

The fresh buffy coats (Leukocyte Source) from local blood bank (Anhui blood center) were used in this study as the source of peripheral blood mononuclear cells (PBMCs) for NK cells expansion. The PBMCs were isolated from the buffy coats by using Ficoll-Paque (Biosharp, Anhui, China). A modified feeder-free human NK cells expansion system, as described previously, was used for NK cell expansion [31,32]. In brief, 6 × 10^7^ PBMCs were cultured in KBM-581 medium (Corning, 88-581-CM) supplemented with 5% of heat-inactivated autologous plasma, 1000 IU/mL rhIL-2 (Jinsili, Jiangsu, China) and anti-CD3 monoclonal antibody (eBioscience, San Diego, CA, USA), in an anti-CD16 monoclonal antibody (Beckman Coulter, Inc., Brea, CA, USA) immobilized culture flask. The cultures were continued to culture for 17–27 days at 5% CO_2_, 37 °C, by adding fresh medium every 2–3 days to keep the cell density between 1.8 and 3.2 × 10^6^ cells/mL until the desired cell number was reached. Total cell numbers were counted using trypan blue by an automated cell counter (Countstar, Shanghai, China). To determine the percentage of NK cells, cells were stained for CD3 and CD56, followed by flow cytometry analysis. The final cell qualified indicators included proportion of living cells ≥90% and proportion of CD56^+^ cells ≥80%.

### 2.3. Analysis of Receptors Expression Level of eNKs

After 17–21 days cultured, eNKs were harvested and washed twice with PBS to remove culture medium. Cells resuspended in PBS containing 10% mouse serum (Future, Gangzhou, China, F001008) at 4 °C for 15 min (in order to block Fc receptors), prior to incubation with appropriate antibodies at 4 °C for a further 30 min. For the detection of intracellular cytokine, such as perforin, Granzyme B and IFN-γ, cells were pretreated with 2.5 ng/mL monensin (Sigma, St. Louis, MO, USA. 22373-78-0) for 4 h. The Foxp3/Transcription Factor Staining Buffer Set (q, 00-5523-00) was used as instructed. All antibodies were purchased from Biolegend, San Diego, CA, USA. DAPI (10 μg/mL) was used to differentiate dead cells at 5 min before test. Then 13-color FACS analysis of NK marker was performed on a flow cytometer cytoflex (Beckman Coulter, Inc., Brea, CA, USA).

### 2.4. Antibody

The following fluorescently conjugated antibodies were used for phenotypic analysis of NK cells: anti-LFA-1 (363404); anti-CXCR3 (353720); anti-PD-1 (329906); anti-CD107a (328612); anti-NKP44 (325116); anti-CD158e1 (312706); anti-CD158d (347006); anti-CD27 (356412); anti-CCR4 (359412); anti-DNAM-1 (338316); anti-CD16 (302040); anti-Granzyme B (515406); anti-CD62L (304806); anti-CD69 (310910); anti-NKP80 (346706); anti-NKP30 (325210); anti-CD158f (341304); anti-CD158b (312612); anti-Tim-3 (345012); anti-CD94 (305504); anti-TIGIT (372706); anti-TRAIL (308206); anti-CD57 (322306); anti-CX3CR1 (341610); anti-NKG2D (320808); anti-Perforin (353310); anti-Ki67 (350504); anti-IFN-γ (506518); anti-CD94 (305506); anti-NKp46 (331916); anti-CTLA4 (369614); anti-CD96 (338416); anti-41BB (309818); anti-CD25 (356108) were purchased from Biolegend. anti-CD159a/NKG2A (FAB1059P) and anti-NKG2C (FAB138G) were purchased from R&D. 

The following fluorescently conjugated antibodies were used to identify immune cell types in eNK or PBMC: anti-human Lineage Cocktail (348803); anti-CD56 (362550); anti-CD3 (300430); anti-CD33 (366620); anti-HLA-DR (307606); anti-CD14 (301836); anti-CD19 (302242); anti-CD11b (301322); anti-CD25 (356108); and anti-FOXP3 (320108) were purchased from Biolegend, San Diego, CA, USA.

### 2.5. eNKs Cytotoxicity Assay

CD3^−^CD56^+^NK cells and CD56^+^ cells were purified from eNKs by using human CD3^+^ MicroBeads (Miltenyi biotec, 130-050-101) and/or human CD56^+^ MicroBeads (Miltenyi biotec, 130-050-401) following manufacturer’s instruction. Briefly, 10^6^ PBMC or eNKs, resuspended in 80 µL of buffer, were incubated with 20 µL of MicroBeads for 15 min at 4 °C. Then, interested cells were purified with magnetic separation columns. 

#### 2.5.1. Flow-Based Killing Assay

Firstly, 10^6^ of K562 and Molt4 cells were harvested and suspended in RPMI 1640 (Hyclone, SH30809.01) containing 2% fetal bovine serum (Biological Industries, Cromwell, CT, USA, 04-001-1 ACS), incubated with 5 μM CFSE (Biolegend, San Diego, CA, USA, 423801) in the dark at 37 °C for 15 min, followed by 3 times of wash to remove CFSE. Then, cell resuspended in RPMI 1640 containing 10% FBS. Collected eNKs, purified CD3^−^CD56^+^ NK cells or purified CD56^+^ cells were suspended at indicated density in RPMI 1640 containing 10% FBS, incubated with K562 cells at 37 °C for 3.5 h. Propidium iodide solution (Biolegend, 421301) was added for 5 min to identify dead cells at room temperature before test.

#### 2.5.2. RTCA-Based Killing Assay

This assay was performed on real time cell analysis (ACEA Biosciences, San Diego, CA, USA) according to manufacturer’s instructions. Briefly, A375, Ho8910 or Skov3 cells washed twice and resuspended in RPMI 1640 contain 10% FBS, added 1.6 × 10^4^ cells/well/100 μL, and cultured in constant temperature incubator at 37 °C for about 13 h. eNKs washed twice and resuspended in RPMI 1640 medium containing 500 IU rhIL-2 (Jiangsu Kingsley Pharmaceutical Co., Ltd., Jiangsu, China) and 10% FBS. CD56^+^ cells co-incubation with A375, Ho8910, or Skov3 cells in constant temperature incubator at 37 °C for 4 h.

### 2.6. Analysis of the Distribution of eNKs in NCG Mice

eNKs were harvested after 17–21 days cultured, and an automatic cell counter (Countstar, Shanghai, China) was used to adjust the cell density to an appropriate density. Female NCG mice received 1 × 10^7^/250 μL eNKs. Mice were also intraperitoneally injected with rhIL-2 (50,000 IU/mice) every other day. 

At the time of sacrifice, tissues such as spleen, liver, and lung were taken, and an automatic tissue grinder (Miltenyi Biotec, Bergisch Gladbach, Germany) was used according to manufacturer’s instructions. Briefly, spleens were placed in 5 mL PBS and crushed with the m_spleen_01 program. Lungs were cut into 1–2 mm^3^ pieces and digested in RPMI 1640 with 1 IU/mL DNase I (Sigma, D5025) and 10 µg/mL collagenase IV (Sigma, St. Louis, MO, USA, C5138) in a constant temperature incubator at 37 °C for 1.5 h, followed by crushing with the m_lung_01 program. Liver was placed in 5 mL RPMI 1640 with 1 IU/mL DNase I and 10 µg/mL collagenase IV, following by being digested and crushed with the 37 °C_m_LDK_01 program. An injector was used to flush out the bone marrow cells. Mouse peripheral blood was lysed with red blood cell lysis buffer (Solarbio, Beijing, China, R1010) for 5 min at room temperature. The peritoneal fluid of mice was obtained by irrigation with PBS. The single-cell suspensions were washed twice and resuspended in PBS containing 10% mouse serum at 4 °C for 15 min, and incubated with antibodies at 4 °C for 30 min. Cell staining with the following antibody: anti-human FITC-CD3 (300406), anti-human-PE/Cy7-CD56 (318318), anti-human-APC/Cy7-CD45 (368516), and anti-mouse-PerCP/Cy5.5-CD45 (103132). All antibodies were purchased from Biolegend. 

### 2.7. Tumor Mouse Models

NCG mice were used when more than 6 weeks of age. All mice were randomly applied to any experimental group. For the imaging experiment to examine co-localization of eNKs and tumors, female NCG mice received 2 × 10^6^ Skov3-luc/mice through hypodermic injection on day 14. eNKs were stained with 3.5 μg/mL DIR (1,1′-dioctadecyl-3,3,3′,3′-tetramethylindotricarbocyanine iodide, Invitrogen^®^M, Carlsbad, CA, USA, D12731) in the dark at 37 °C for 30 min, then washed 3 times before injection. For the anti-tumor experiment of eNKs, NCG received 4 × 10^6^ Skov3-luc/mice or 8 × 10^5^ Ho8910-luc/mice through intraperitoneal injection on the indicated days. eNKs were injected intravenously into the tail vein after tumor cell infusion, and were supported by the injection of rhIL-2 every other day.

For imaging experiments, mice were injected intraperitoneally with D-luciferin (150 μg/g) in PBS and imaged using the bioluminescence imaging system (IVIS Spectrum, PerkinElmer, Boston, MA, USA) according to the manufacturer’s instructions to monitor tumor burden. For DIR imaging experiments, the fluorescent signal was acquired with a 750 nm emission filter at the same time point as the bioluminescence imaging.

### 2.8. Histopathological Evaluation and Blood Analysis

Tissues were fixed in 10% neutral-buffered formalin (NBF) for about 24 h, followed by dehydration, embedded in paraffin blocks, and then cut into 5 μm sections, which were stained with hematoxylin-eosin (H&E). Images were obtained using the ZEISS Axioskop2 plus advanced positive microscope.

Mice blood was collected in EDTA-coated tubes and analyzed on an automatic blood analyzer (SYSMEX, Tokyo, Japan, XT-1800i) according to manufacturer’s instructions. Serum was collected and tested by automatic biochemical analyzer according to manufacturer’s instructions.

### 2.9. Statistical Analysis

Before statistical analyses, data were tested for normal distribution (D’Agostino-Pearson omnibus normality test). If data were not normally distributed, statistical analyses were performed in Statistical Product and Service Solutions (IBM SPSS Statistics 22, Chicago, IL, USA.). The comparisons were made using Wilcoxon signed rank test. If data were normally distributed, statistical analyses were performed in Prism software (Graphpad 7.0). The comparison was performed using unpaired or paired two-tailed *t*-tests, and one-way ANOVA tests. Linear regressions were evaluated with Pearson correlation tests. The survival curve was analyzed using the log-rank (Mantel–Cox) test. All data are presented as mean ± S.D, and the “n” values in each figure legends represent the number of the donor. Significant differences were indicated for each figure and defined as ns: non-significant, *p* > 0.05; *, *p* < 0.05; **, *p* < 0.01; ***, *p* < 0.001, and ****, *p* < 0.0001.

## 3. Results

### 3.1. Expansion of PBMC-NK Ex Vivo Yields a High Quantity of High Purity NK Cells with Enhanced Cytotoxicity

**Figure 1:**
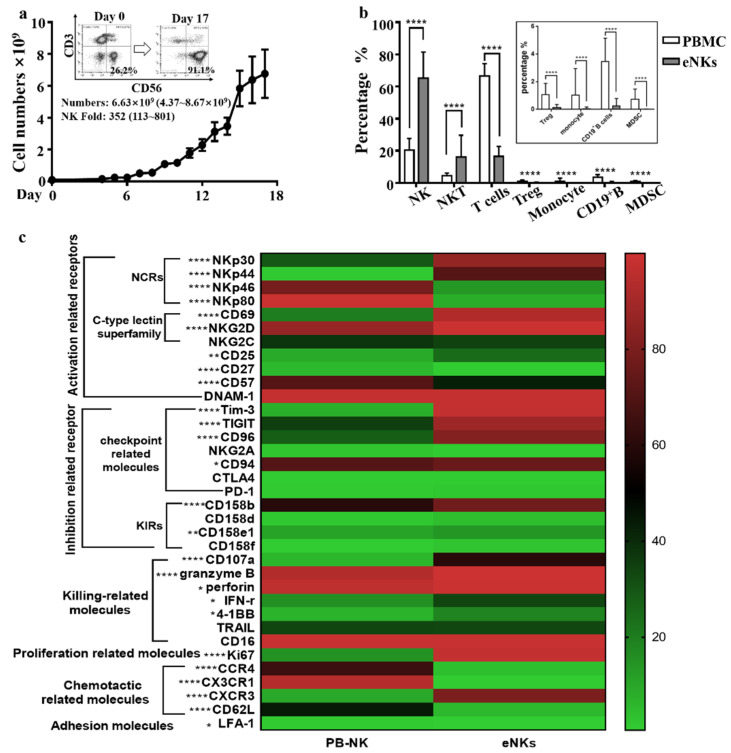
NK cells with enhanced cytotoxic activity were expanded ex vivo. PBMCs were isolated and cultured using a feeder cell-free NK cell expansion system. After 15 to 17 days of culture, expanded cells were harvested and identified. (**a**) A curve of the total number of expanded cells was generated for the expansion period. Data are shown as mean ± SD (*n* = 32). Inset images show the purity of NK cells before (Day 0) and after (Day 17) expansion from one representative experiment. (**b**) The proportion of different types of immune cells before and after PBMC expansion were determined for comparison by two-tailed paired Student *t*-test (*n* = 28). (**c**) Heat map showing the expression levels of surface receptors and intracellular granule components in CD3^−^CD56^+^ NK cells before and after expansion (*n* = 31). Significance was determined by Wilcoxon signed-rank test. * *p* < 0.05; ** *p* < 0.01; **** *p* < 0.0001.

In this study, we investigated the use of a feeder cell-free human NK cell expansion system for increasing the number and activation of NK cells ex vivo without the need for NK cell purification and feeder cell co-culture. After 15 to 17 days of culture in this system, 6.76 ± 4.31 × 10^9^ total cells were harvested (Figure 1a). The mean proportion of CD3^−^CD56^+^ NK cells increased from 20.38 ± 7.28% (range: 9.01–39.73%) to 75.22 ± 16.34% (range: 33.9–93.16%), while the proportion of CD56^+^ cells increased from 24.89 ± 7.57% (range: 11.28–45.33%) to 86.9 ± 5.94% (range: 71.3–95.29%). By contrast, the proportion of CD3^+^CD56^−^ T cells decreased from 66.45 ± 7.84% to 16.52 ± 6.20% after expansion. CD14^+^ monocyte, CD19^+^ B cells, and immunosuppressive cells (e.g., CD3^+^CD4^+^CD25^+^Foxp3^+^ Treg cells and lineage-HLA-DR^-^CD33^+^CD11b^+^ MDSC) were almost undetectable among the expanded cells (Figure 1b). These results indicated that a large number of highly pure NK cells could be obtained from PBMC cells after ex vivo culture.

Given that different ex vivo expansion systems have been shown to affect the expression levels of various activating and inhibitory receptors in NK cells [33], we thus compared the expression of surface receptors of expanded natural killer cells (eNKs) with that of PB-NK cells. The results showed that the levels of several activation-related receptors (e.g., NKp44, NKp30, NKG2D, etc.) were upregulated after NK cell expansion (Figure 1c and Figure S1). However, some checkpoint-related receptors (e.g., TIGIT, Tim3, etc.), and KIRs (e.g., CD158b and CD158e1) were also significantly upregulated after NK expansion (Figure 1c and Figure S1), which suggested that a combination with checkpoint antibody strategy would enhance the efficacy of the eNKs. This discrepancy was different from that observed in previously reported expansion systems [34,35]. Notably, multiple cytotoxic effector-related molecules (i.e., CD107a, granzyme B, perforin, IFN-γ, and 4-1BB) were significantly upregulated in eNKs compared to their expression in PB-NKs (Figure 1c), which suggested that NK cells could show enhanced anti-tumor cytotoxicity following ex vivo expansion. 

Immunofluorescent staining with flow cytometry analysis revealed that three main cell subsets were present among the expanded cells: CD56^−^, CD3^+^CD56^+^ NKT, and CD3^−^CD56^+^ NK cells (Figure 1b). To determine which cell group exerted the strongest cytotoxic activity, we first collected CD56^+^ cells by depleting CD56^−^ with microbeads. Microbead-selected CD56^+^ cells showed no significant difference in their killing of K562 cells with that of the same quantity of non-selected CD56^+^ cells (Supplementary Materials Figure S2a), which suggested that CD56^+^ cells were the principal cell type to mediate tumor cell killing. We then compared the cytotoxicity of selected CD3^−^CD56^+^ NK cells with that of the same number of CD3^−^CD56^+^ cells in microbead-selected CD56^+^ cells and found that the microbead-selected CD56^+^ cells exhibited significantly higher cytotoxicity than the selected CD3^−^CD56^+^ NK (Figure S2b), which suggested that CD3^+^CD56^+^ NKT cells also contribute anti-tumor activity. Moreover, the purity of CD56^+^ cells in eNKs was correlated with their anti-K562 cytotoxicity (Figure S2c). We therefore hypothesized that the anti-tumor effectors among the eNKs were mainly CD56^+^ cells.

**Figure 2:**
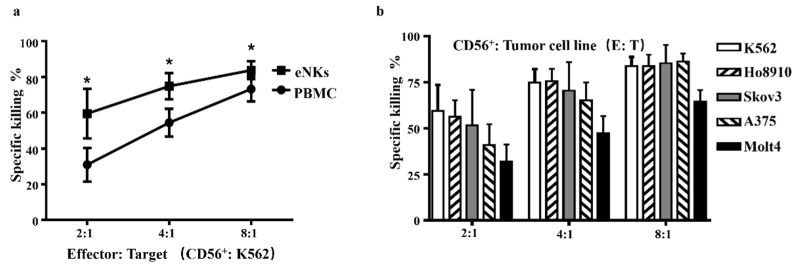
eNK enhanced cytotoxicity against multiple cancer cell lines ex vivo. (**a**) Comparison of the K562 cell killing activity by equal CD56^+^ cells numbers of non-selected PBMC CD56^+^ cells (determined by flow cytometry) before and after expansion. (**b**) eNK killing activity against different cancer cell lines. Results are representative of at least five independent experiments. Data were analyzed by two-tailed paired Student *t*-test. Data are represented as means ± SD. * *p* < 0.05.

To determine whether the cell killing activity of CD56^+^ cells was altered during the expansion process, we next compared the anti-tumor activity of PB-NK cells before and after expansion. The results showed that after expansion, eNKs had significantly higher cytotoxicity against K562 cells ex vivo (Figure 2a). These data indicated that NK anti-tumor cytotoxicity was enhanced following expansion by this system. We then tested the anti-tumor activity of eNKs towards different human tumor cell lines ex vivo and observed that eNKs exhibited effectiveness in killing a variety of tumors (Figure 2b). In addition, we also observed that eNKs exhibited similar cytotoxicity against Ho8910 and Skov3 to that in the K562 cell line (Figure 2b), which indicated that eNKs were sensitive toward OC killing.

### 3.2. Proliferation, Lifespan, and Tumor Co-Localization Were Enhanced in eNKs in Ovarian Tumor-Bearing Mice

**Figure 3:**
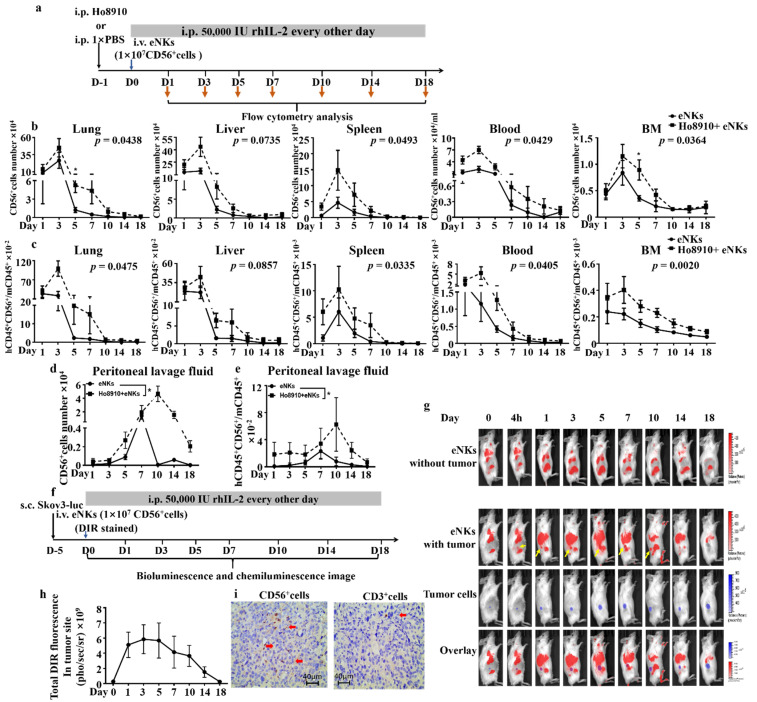
eNKs showed enhanced proliferation, lifespan, and tumor co-localization in xenograft ovarian tumor-bearing NCG mice. (**a**) eNKs were adoptively transferred to ovarian tumor- or non-tumor-bearing NCG mice. (**b**) Total human CD56^+^ cell numbers were determined by automatic cell counter at the indicated times. (**c**) Flow cytometry analysis of the persistence and distribution of adoptively transferred CD56^+^ cells in lung, liver, bone marrow, spleen, and blood. (**d**,**e**) Flow cytometry and automatic cell counter analysis of the percentage and total human CD56^+^ cells in mouse peritoneal lavage fluid after cell injection at the indicated times. (**f**) Experimental design and timeline of Skov3-luc injection and eNK co-localization assays. (**g**) Bioluminescence and chemiluminescence imaging showing the tumor and transferred eNKs localization at the indicated times. (**h**) DIR signal was quantified via chemiluminescence at the indicated times. (**i**) Immunohistochemical imaging of human CD56^+^ and CD3^+^ cells in tumor tissue. Results are representative of at least three independent experiments. All data were analyzed by two-tailed paired Student *t*-test. Data are represented as mean ± SD. * *p* < 0.05.

Migration, proliferation, and survival by NK cells in the tumor environment are well-established as essential factors that contribute to their anti-tumor efficacy in vivo. Thus, we infused Ho8910 tumor-bearing and non-tumor-bearing NCG mice with eNKs, then isolated multiple organs and analyzed the eNKs by flow cytometry over an 18-day time period (Figure 3a). In vivo proliferation kinetics of the adoptively transferred cells in NCG mice showed that eNKs primarily localized to lung and liver tissue, followed by spleen, blood, and bone marrow (Figure 3b), with the ratio of human CD45^+^CD56^+^ cells to mouse CD45^+^ cells peaking at day 3, and remaining stable for approximately 10–14 days in vivo. Notably, in all five tissue types, we fund that the abundance of adoptively transferred eNKs peaked at the same time in both two groups; however, a higher percentage and number of human CD45^+^CD56^+^ cells in tumor-bearing mice than in non-tumor-bearing mice (Figure 3b,c) was observed, which suggested that the tumor microenvironment may positively affect proliferation of adoptively transferred eNKs.

To further examine whether the tumor microenvironment affects the persistence of eNKs in vivo, we next collected peritoneal lavage fluid from the Ho8910 tumor-bearing mice and non-tumor-bearing mice and quantified the abundance of CD45^+^CD56^+^ cells. Both the number of human CD45^+^CD56^+^ cells and the ratio of human CD45^+^CD56^+^ cells to the mouse CD45^+^ cells were significantly higher in tumor-bearing mice than that in non-tumor-bearing mice (Figure 3d,e), both of which reached their highest values at day 7 in non-tumor-bearing mice, but peaked at day 10 in tumor-bearing mice (Figure 3c,e). In addition, the CD45^+^CD56^+^ cells returned to baseline level in non-tumor-bearing mice after 18 days of eNKs injection. However, a high level CD45^+^CD56^+^ cells number in tumor-bearing mice still remained (Figure 3d). These results suggested that the tumor microenvironment would promote eNK survival and proliferation.

In addition, we used bioluminescence and chemiluminescense imaging to investigate whether the adoptively transferred eNKs migrated to the tumor site (experimental schematic in Figure 3f). The results indicated that DIR-stained eNKs indeed migrated to the tumor site within 4 h after infusion (Figure 3g). Moreover, total DIR fluorescence increased at the tumor site from day 0 to day 5 (Figure 3h). Immunohistochemical microscopy showed that the vast majority of eNK cells that infiltrated the tumor tissue were CD56^+^, compared to negligible representation by CD3^+^ cells following cell infusion (Figure 3i), which suggested that CD3^−^CD56^+^ NK cells were the primary immune cell type to migrate and infiltrate into the solid tumor tissue. Collectively, these results indicated that eNK cell migration to the tumor site, lifespan, and proliferation were all enhanced in the tumor microenvironment after infusion.

### 3.3. eNKs Effectively Reduced Ovarian Ascites and Tumor Burden in Ovarian Tumor-Bearing Mice

**Figure 4:**
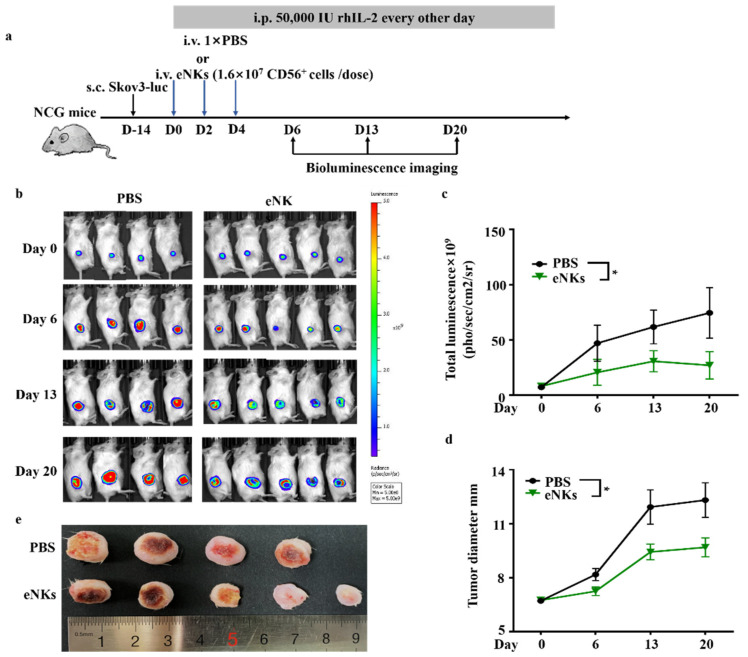
eNKs inhibited tumor growth in a Skov3 xenograft solid tumor model of human OC. (**a**) Schematic timeline of eNK treatment and establishment of a human OC solid tumor model by subcutaneous injection with Skov3-luc cells in NCG mice. (**b**) Animal bioluminescence imaging of the tumor burden following adoptive transfer of eNKs at the indicated times. (**c**) Summary of bioluminescence measurements in different treatment groups over the 20-day experimental period. Significance was determined by two-tailed paired Student *t*-test. (**d**) Tumor diameters were measured for each treatment group. Significance differences were determined by two-tailed paired Student *t*-test. (**e**) The images of tumors obtained from the eNK- and PBS-treated (control) groups at the 20th day after the initial eNK infusion. Results are representative of two or three independent experiments. Data are represented as means ± SD. * *p* < 0.05.

In light of our findings that eNKs exhibited OC cell killing ex vivo, and that they could persist and proliferate in the tumor microenvironment, we next explored whether infusion with eNKs could alleviate the tumor burden in vivo. First, we established a solid tumor model of human OC by hypodermic injection of Skov3-Luciferase (Luc) cells into NCG mice. Based on our observations of the in vivo distribution and persistence of eNKs, we designed an experimental regimen including three total infusions administered in two-day intervals (Figure 4a). This treatment plan resulted in the retention of a high number of NK cells in mice for at least 7 days after the initial intravenous injection. In addition, 50,000 IU rhIL-2/mice was intraperitoneally injected every other day to facilitate human NK cell prolonged retention. Tumor development was detected by bioluminescence imaging and tumor volume measurement, weekly (Figure 4b–d). The results confirmed that the therapeutic adoptive transfer of eNKs led to obvious inhibition of tumor development. At 28 days after the first infusion, average tumor diameter in the treatment group was also significantly lower than that in the control mice (Figure 4e). These results together illustrated the efficacy of the adoptive transfer of eNKs as a therapeutic treatment for solid ovarian tumors.

**Figure 5:**
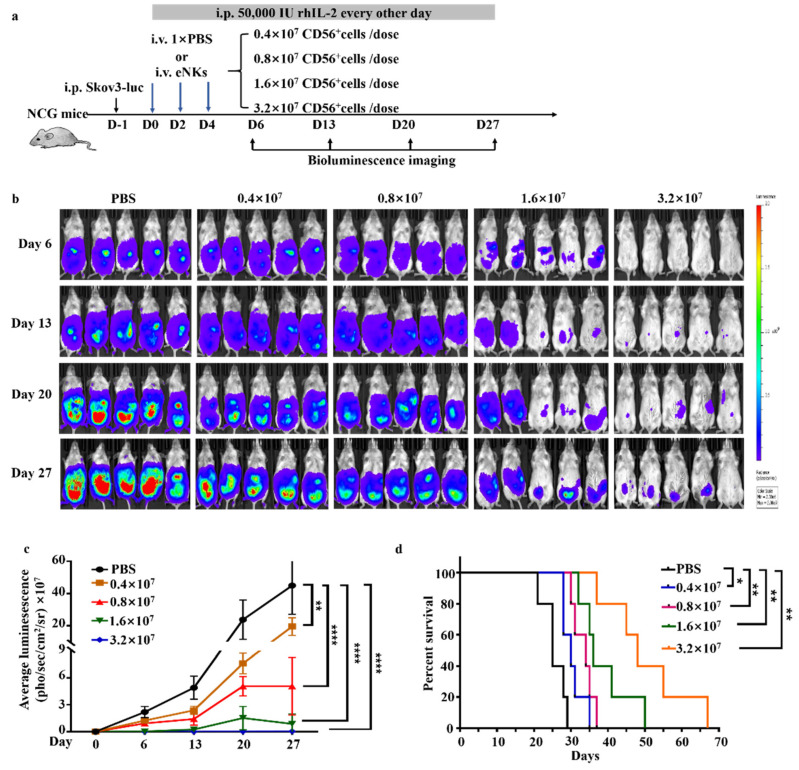
Adoptive transfer of eNKs led to reduced tumor burden and improved survival in a Skov3 xenograft peritoneal metastasis model of human OC. (**a**) Experimental timeline for adoptive transfer of different doses of eNKs for treatment of human metastatic OC tumors in an abdominal cavity model established by intraperitoneal injection of Skov3-luc cells into NCG mice. (**b**) Animal bioluminescence imaging was used to quantify tumor burden after eNK transfer at the indicated times. (**c**) Summary of bioluminescence measurements for group treated with different eNK doses over the 27-day experimental period. (*n* = 5 mice per group in one experiment). Significant differences were determined by one-way ANOVA. (**d**) Mouse survival compared across groups and analyzed using the log-rank test. Significant differences were determined by analysis of variance (ANOVA). Data are represented as means ± SD. * *p* < 0.05; ** *p* < 0.01; **** *p* < 0.0001.

Ovarian tumors are commonly understood to extensively metastasize to the abdominal cavity. In order to assess the efficacy of eNKs against metastatic abdominal ovarian tumors, increasing dosages of eNKs, ranging from 0.4 × 10^7^ to 3.2 × 10^7^ CD56^+^ cells/dose with a total of three doses every other day, were administrated to xenograft NCG mice at one day post-intraperitoneal injection of Skov3-luc OC cells. Bioluminescence imaging of these mice revealed that eNK infusion led to obvious inhibition of ovarian tumor growth in the abdominal cavity, which increased in an apparently dose-dependent manner (Figure 5b). In addition, the survival rates in the eNK treatment groups were significantly higher than that in the PBS-infused control mice (Figure 5c). Similar results were also obtained in a Ho8910-luc mouse model (Figure S3). These data demonstrated that eNKs could effectively inhibit abdominal metastases of ovarian tumors in vivo.

The development of OC in patients is usually accompanied by the occurrence of ascites [36], which provides a microenvironment that is conducive to tumor cell growth and suppression of immune effector function (including that of NK cells). Thus, we investigated the development of ascites in an OC-engrafted mice model at day 28 following infusion with eNKs. The results showed that eNK treatment reduced abdominal circumference in a dose-dependent manner (Figure S3e). Comparison of ascites volume between eNK treatment groups and PBS controls further demonstrated the potential of eNK treatment in reducing ascites (Figure S3f).

**Figure 6:**
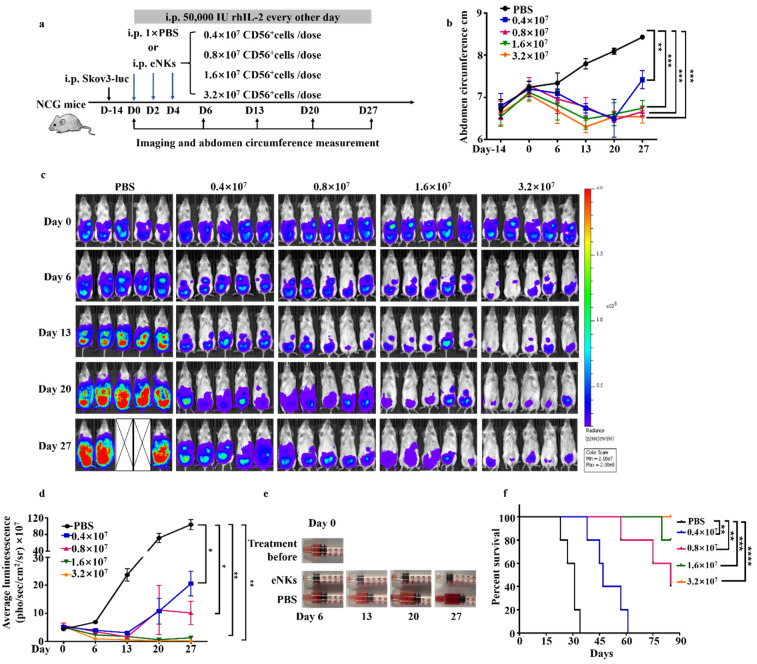
eNKs reduced the ascites burden and improved survival rates in a Skov3 xenograft model of human OC. (**a**) Experimental design for intraperitoneal adoptive transfer of eNKs into xenograft mice beginning 2 weeks following injection of Skove3-luc. (**b**) Abdominal circumference was quantified for each group at the indicated times (*n* = 5 mice per group from one experiment). (**c**) Bioluminescence imaging was used to quantify tumor burden after eNK transfer over 27 days. (**d**) Summary of bioluminescence measurements for each group at the indicated times (*n* = 5 mice per group in one experiment). Significant differences were determined using one-way ANOVA. (**e**) Images showing differences in ascites volume between eNK- and PBS-treated groups at the indicated times. (**f**) Mice were monitored for Kaplan–Meier survival curve, and significance was determined by log-rank test. Data are represented as means ± SD. * *p* < 0.05; ** *p* < 0.01; *** *p* < 0.001, **** *p* < 0.0001.

We then sought to determine whether eNKs could function in reducing the burden of ascites in OC. To this end, we established a Skov3 xenograft model for a human OC model (schematic in Figure 6a), and at roughly 2 weeks after Skov3-luc cell injection, we found that the abdominal circumference of mice increased due to ascites formation (Figure 6b). Comparison of the tumor and ascites burden between animals treated by adoptive transfer of eNKs and the PBS control group showed that both the tumor and ascites burden were reduced in OC mice treated with eNKs compared to controls, and that these mice had longer survival times (Figure 6b–f). The same results were obtained using a Ho8910-luc mouse model (Supplementary Materials Figure S3a–d). These results demonstrated that eNKs exhibited anti-tumor activity in an OC model and also reduced the ascites burden. Furthermore, we adoptively transferred eNKs to the mouse when the tumor development in later stages with the ascites load was severe (Figure S4e–h). In Figure S4h, the results shown that the abdominal cavity of mice had severe ascites load at day 0 before NK cells therapy, but the ascites disappeared after eNK cell infusion at Day 6 and Day 13. Nevertheless, the tumor burden did not show the similar reduction as ascites (Figure S4g). These results suggesting that NK cells could kill the tumor cell more efficiently and rapidly in abdominal cavity fluid than in tumor mass.

### 3.4. Safety Evaluation of eNKs In Vivo

**Figure 7:**
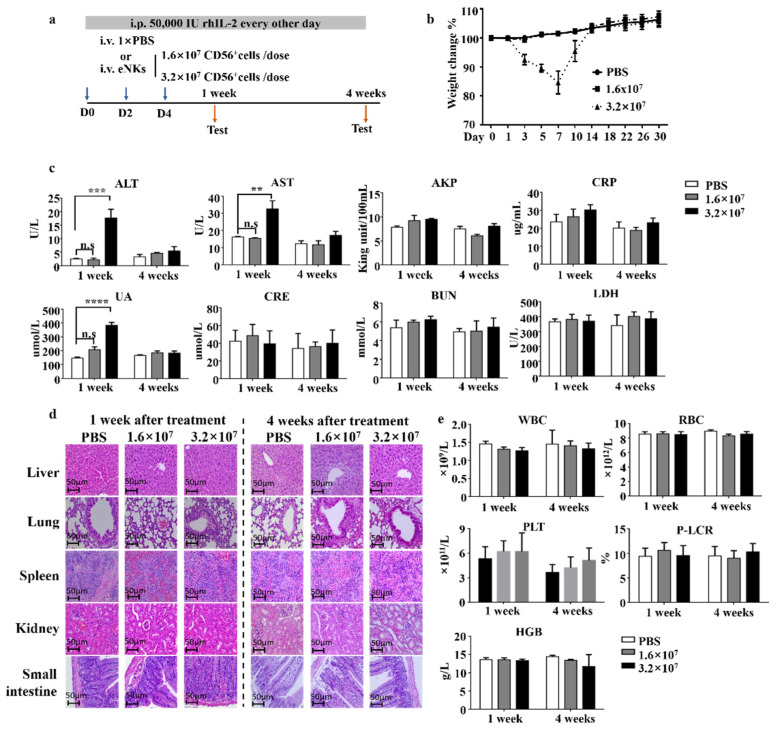
Safety evaluation of eNKs in NCG mice. (**a**) eNKs were adoptively transferred to non-tumor-bearing NCG mice. (**b**) Weight loss was recorded in mice following the injection with two doses of eNKs or PBS, as described in (A). (*n* = 5) (**c**) Serum samples were collected after 1 or 4 weeks and then analyzed for standard biochemical parameters: alanine aminotransferase (ALT), aspartate aminotransferase (AST), alkaline phosphatase (AKP), C reactive protein (CRP), uric acid (UA), creatine (CRE), blood urea (BUN), and lactic acid dehydrogenase (LDH). (**d**) Representative images of immunohistochemical staining of GvHD-targeted tissues (including liver, lung, small intestine, spleen, and kidney) at the indicated times. (**e**) Blood test analysis at the indicated times after eNK treatment. White blood cells (WBC); red blood cells (RBC), platelet (PLT), platelet to larger cell ratio (P-LCR), and hemoglobin (HGB). Results are representative of two independent experiments. Data were analyzed by two-tailed paired Student *t*-test. Data are represented as means ± SD. ** *p* < 0.01; *** *p* < 0.001, **** *p* < 0.0001.

In our expansion system, the expanded cell products were not purified prior to infusion, which thus resulted in an unavoidable but modest proliferation of T cells during the NK expansion (Figure 1b). To evaluate the safety of eNKs, we established a mouse model for adoptive cell transfer schematic in Figure 7a. We examined the effects of two doses of CD56^+^ cells (1.6 and 3.2 × 10^7^ cells/dose) in NCG mice (Figure 3a) and found that the high dose group exhibited reduced mobility, tachypnea, and approximately 15% weight loss in the first week of treatment compared to control animals (Figure 7b). After the first week, behavior gradually returned to normal and weight loss reverted to that recorded at the start of treatments. In addition, in the first week, alanine aminotransferase (ALT) and aspartate aminotransferase (AST) levels were significantly elevated in the high treatment group compared with those of the PBS-treated controls, which suggested the possibility of acute liver injury. In addition, we observed elevated uric acid (UA) levels in the high dose group, which indicated abnormal kidney metabolic function (Figure 7c). Notably, all three indicators returned to normal after 4 weeks of treatment. Moreover, we detected no obvious damage to the GvHD-targeted tissues (i.e., liver, lung, small intestine, spleen, or kidney) (Figure 7d).

Taken together, these results suggested that the observed toxicity in the high-dose group was temporary, and no parenchymal damage was incurred to mice. GvHD-related symptoms can include anemia, increased white blood cells, and other issues [37]. Blood tests indicated neither the occurrence of anemia nor an increase in white blood cell counts in either eNK dosage group (Figure 7e). In addition, we observed no death of animals in either treatment group until 6 months after the experiment’s initiation. Cumulatively, these results indicated that high doses of eNKs could induce short-term toxicity, but not irreversible damage or GvHD in mice. However, although the infusion dose to 3.2 × 10^7^ CD56^+^ cells/dose/mouse (maximal dose of 4 × 10^7^ cells/dose/mouse, total three doses) does not exactly match with the GvHD reaction, it is most likely a potentially toxic dose that could cause GvHD due to it containing a high dose of T cells in final product. Thus, these results demonstrated that contaminated T cells in eNK could induce GvHD when the infusion dose was increased to 4 × 10^7^ cells/dose/mouse (three doses), which contained 2.4 × 10^7^ T cells/mouse in total.

## 4. Discussion

Several previous studies have shown that adoptively transferred NK cells can effectively increase the cell number and function of NK cells in tumor patients [3,5,38]. In particular, high cytotoxicity and specificity, as well as high infiltration and subsequent persistence in tumor sites, are all essential requirements for the effective killing of solid tumors by adoptively transferred NK cells. Here, we demonstrated that NK cells expanded in an ex vivo feeder cell-free system could migrate to the tumor site to effectively inhibit ovarian tumors and ascites formation in a xenograft murine model.

NK cells obtained from different ex vivo expansion systems (such as those using a combination of cytokines, cytokines coupled with antibodies, or membrane particles coupled with cytokines/antibodies to stimulate NK cell expansion) exhibit distinct phenotypes which are correlated with their anti-tumor potency ex vivo and in vivo [3,35,39]. In this study, we analyzed a series of eNK cell phenotypes and found that molecules related to cell killing (e.g., CD107a, granzyme B, perforin, IFN-γ, and 4-1BB) significantly increased after expansion, suggesting enhanced cytotoxicity. Following the in vivo and in vitro experiments, eNK cells showed strong killing activity against tumor cell lines such as K562 in Figure 2a, infiltration ability to tumor site in the tumor bearing mouse model in Figure 3g–i, and the vivo proliferative activity in Figure 3b–e. These data collectively show the advantages of eNK cells. In addition, killer cell immunoglobulin-like receptors (KIRs) (including CD158b and CD158d) were also upregulated after expansion. KIRs restrict the potency of autogenous NK cells through self-histocompatibility antigens. While for allogeneic NKs, particularly human lymphocyte antigen (HLA)-unmatched donors are typically selected, a mismatch occurred between KIR and HLA that enhanced after expansion, and which conferred stronger anti-tumor potency in allogeneic eNKs. eNKs also show upregulation of natural cytotoxicity receptors (NCRs) (NKp30 and NKp44) and NKG2D. Previous studies have reported that human OC cells express high levels of NK cell-activated receptor ligands (NKG2D, NCRs, DNAM-1, etc.) and low levels of NK cell-inhibited receptor ligands (NKG2A/CD94, PD-1, and TIGIT) [14,15]. These findings further suggest an enhancement to the anti-ovarian tumor potency of eNKs after expansion. 

In previous studies, adoptively transferred NK cells have shown limited therapeutic efficacy against OC because their persistence and expansion were restricted in the solid tumor [40,41,42] or ovarian ascites microenvironments [25,26,43,44]. Our data demonstrate that adoptively transferred eNKs could migrate to the tumor site within 4 h after infusion (Figure 3g–h). These eNKs were also found in both higher total quantities and greater proportions with longer persistence in multiple tissues in a tumor-bearing NCG model. These results indicated that infused eNKs could reach the tumor site, sustain activation, and proliferate there with no obvious inhibition by the tumor environment. Notably, although eNKs were not purified prior to infusion, the CD3^−^CD56^+^ NK cell subset exclusively migrated to and infiltrated the tumor (Figure 3i), further confirming that NKs represent the predominant class of expanded immune cells that exert anti-tumor activity.

Adoptively transferred NK cells have previously shown to inhibit OC progression in mouse models [14,27,28,29]. However, their efficacy in ovarian abdominal metastases and ovarian ascites remains unclear. In this study, we demonstrated that adoptively transferred eNKs expanded from donor PBMCs effectively inhibited the progression of OC in both solid tumors and abdominal metastatic tumors in a dose-dependent manner. In addition, these PBMC-derived eNKs could also effectively reduce the existing burden as well as the formation of new ovarian ascites. To test the effects of these eNKs against later stage solid tumors and ascites in vivo, we established a late-stage mouse model of OC with severe tumor and ascites burden. Although we found that eNKs could not inhibit tumor development in this model, they could effectively reduce the burden of ascites. This report thus provides initial data showing that eNKs have clinical value in the treatment of advanced ovarian ascites. In addition, in light of our finding that eNKs mainly localized to the lung and liver after infusion (Figure 3b), further experiments are warranted exploring the potency of eNK cells in the treatment of lung and liver cancer patients with pleural effusion and ascites. 

Other previous studies have reported that NK cell products mixed with T cells may result in GvHD after cell infusion [45,46]. However, there is no evidence reported previously regarding how many T cells would be required to induce GVHD, even in mouse model. It has been reported that infusion of 2 × 10^7^ NK cells/dose/mouse, in which less than 5% of T cells were present, was safe, and no GVHD was observed in mouse models [14,47]. Whereas, our data observed in Figure 7 demonstrated that although our dose was 1.6 × 10^7^ CD56^+^ cells/dose/mouse (equal to 2 × 10^7^ cells/mouse × 3 doses), which contained up to 1.2 × 10^7^ T cells/mouse, it would not induce GvHD. However, when we doubled the infusion cells to 12 × 10^7^ cells/mouse, pathological symptoms including the transient weight loss, elevation of ALT, AST, and UA in the mouse were observed. Indeed, the maximal dose of 12 × 10^7^ cells/mouse (equivalent to 6 × 10^8^ cells/kg in human) is the highest dose of NK cell infusion in clinical trials of both mouse and human reported so far, and it provided important information for eNK on its safety evaluation and guidance for further clinical applications.

## 5. Conclusions

In summary, we used a feeder-free NK cell expansion system to obtain a high quantity of highly pure NK cells from PBMCs ex vivo. eNKs showed enhanced cytotoxicity ex vivo and effectively reduced the tumor burden and ascites in a xenograft OC model. Moreover, infusion with high doses of eNKs did not induce GvHD in mice. Our study demonstrated the efficacy and safety of eNKs for treatment of OC and related ascites, thus providing evidence for their clinical potential.

## Data Availability

The data presented in this study are available from the corresponding author upon reasonable request.

## Supplementary Materials

The following are available online at https://www.mdpi.com/article/10.3390/cancers13225866/s1. The following are the Supplementary data to this article. Figure S1: The expression of NK cells’ marker before and after expansion, Figure S2: CD56+ cells are the principal cell of eNKs to kill tumor cells, Figure S3: eNK treatment resulted in lower tumor burden and improved survival in a Ho8910 xenograft model of human OC, Figure S4: eNKs reduced ascites burden and improved survival in an Ho8910 xenograft model of human OC.
