# Supplementary material for: Anti-Tumor Activity of Expanded PBMC-Derived NK Cells by Feeder-Free Protocol in Ovarian Cancer
Source: Cancers (Basel). 2021 Nov 22;13(22):5866. doi: 10.3390/cancers13225866 (PMC8616155; doi:10.3390/cancers13225866)

Supplementary Materials

Supplement Figures

Figure S1. The expression of NK cells' marker before and after expansion.

The expression of inhibitory checkpoints and NK cell activating receptors before and after NK cells expansion were measured by flow cytometry, and the molecules were indicated in dot plot. PBMC-NK and eNKs-NK indicates the NK cells in PBMC and the expanded NK cell respectively. The dot plots are representative from one donor.

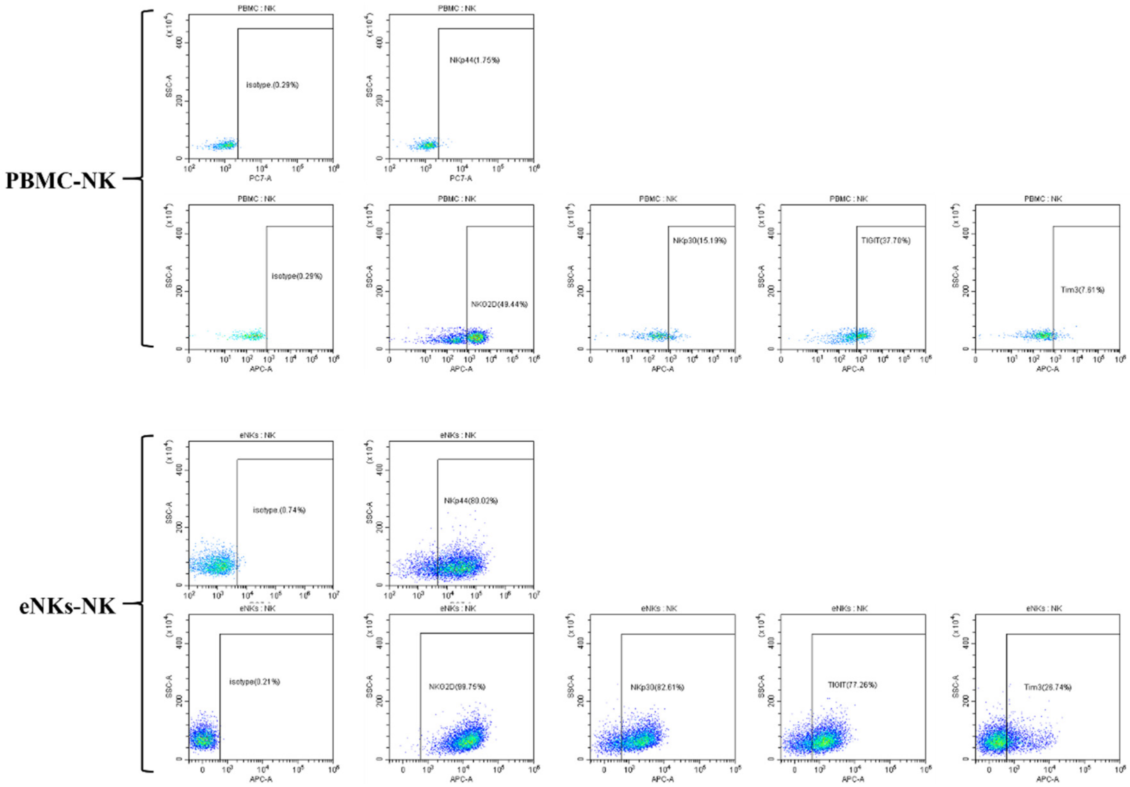

Figure S2. CD56$^+$ cells are the principal cell of eNKs to kill tumor cells.

(**a**) CD56$^+$ cells were selected or non-selected from expansed cells by Microbead kit and their K562 cell killing activity were tested by flow cytometry in equivalent number of CD56$^+$ cells (n = 3 independent experiments). (**b**) CD56$^+$ cells and CD3$^-$CD56$^+$ NK cells were selected from expanded cells by Microbead kit and their cell killing activity towards K562 cells were tested, using equal numbers of NK cells in each group (determined by flow cytometry) (n = 4). (**c**) The efficiency of K562 cell killing by different proportions of CD56$^+$ cells (n = 15). Significance was determined by two-tailed paired Student t test. Data are shown as mean ± SD. * $p < 0.05$; ** $p < 0.01$; **** $p < 0.0001$.

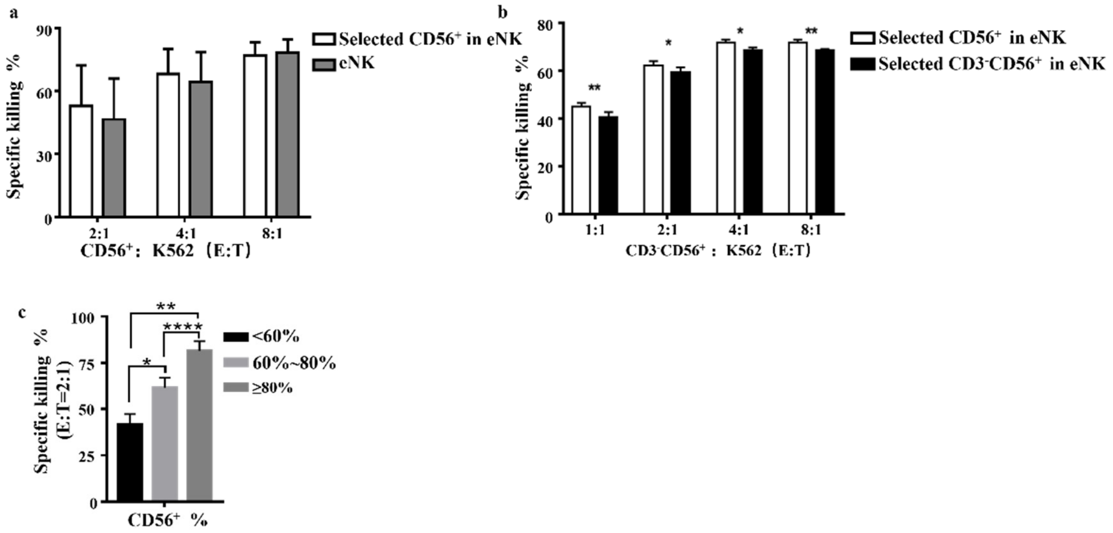

Figure S3. eNK treatment resulted in lower tumor burden and improved survival in a Ho8910 xenograft model of human OC.

(**a**) Experimental design for establishment of a Ho8910 xenograft model of human OC and infusion with several doses of eNKs in NCG mice. (**b**) Bioluminescence imaging of eNK and control animals for quantification of tumor burden after adoptive transfer of eNKs at the indicated times. (**c**) Summary of bioluminescence measurements for each dosage group over the 27-day experiment. Significant differences were determined by one-way ANOVA. (n = 5 mice per group in one experiment) (**d**) Mice were treated as in (**a**), monitored for survival curve, and significant differences were determined by log-rank test. (**e**,**f**) Measurements of abdominal circumference and ascites in tumor-bearing mice showed effective inhibition of tumor-associated ascites development after eNK infusion. Significant differences were determined by one-way ANOVA. Data are represented as means ± SD. * $p < 0.05$; ** $p < 0.01$; *** $p < 0.001$, **** $p < 0.0001$.

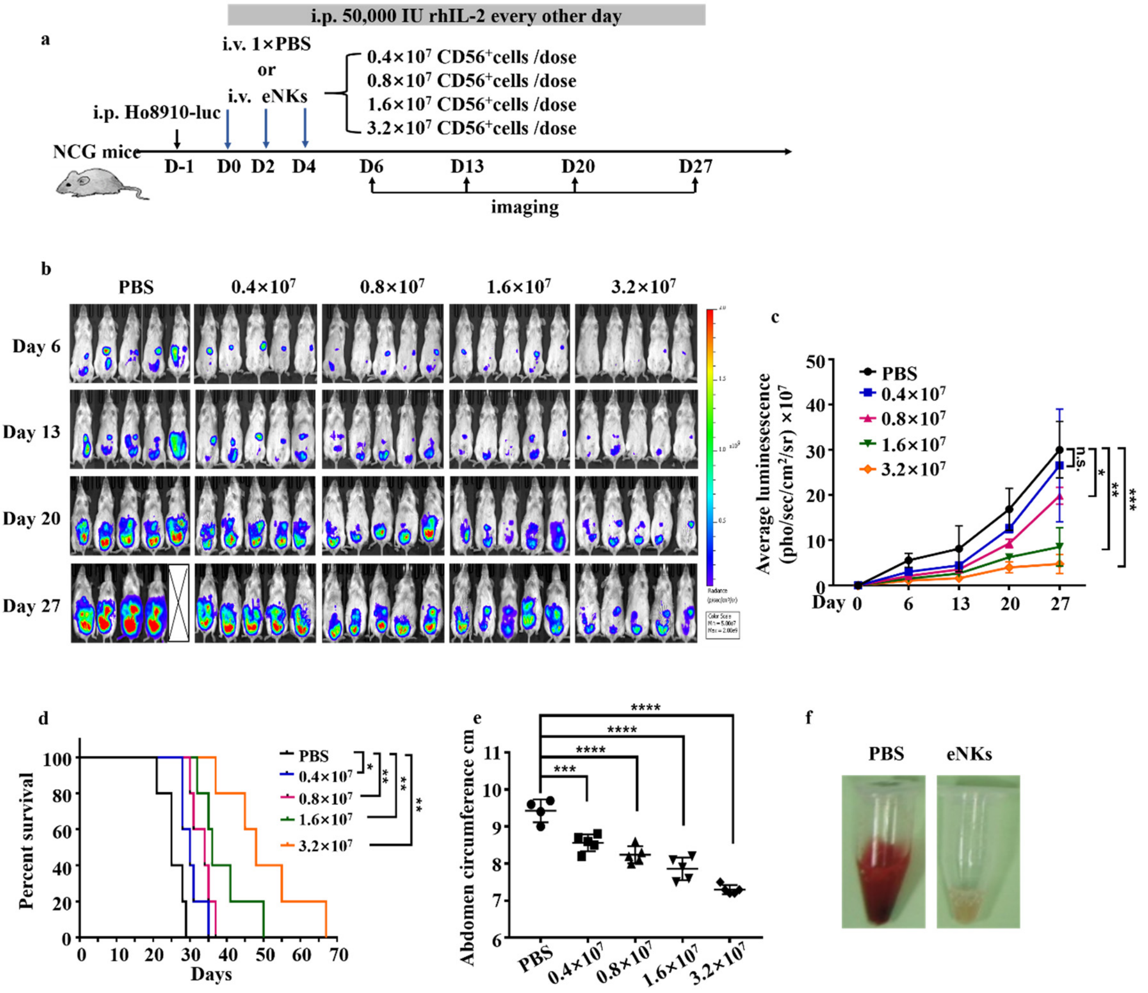

Figure S4. eNKs reduced ascites burden and improved survival in an Ho8910 xenograft model of human OC.

(**a**) Experimental design for intraperitoneal adoptive transfer of eNKs into xenograft mice beginning 2 weeks following injection of Ho8910-luc. (**b**) Bioluminescence imaging was used to quantify tumor burden after eNK transfer over 27 days. (**c**) Summary of bioluminescence measurements for each group at the indicated times (n = 5 mice per group in one experiment). Significant differences were determined using one-way ANOVA. (**d**) Images showing differences in ascites volume between eNK- and PBS-treated groups at the indicated times. (**e**) The schematic presentation of the experimental design. eNKs were adoptively transferred intraperitoneally to xenograft mice beginning 3 weeks following injection of Ho8910-luc injection. (**f**,**g**) Mice were imaged at indicated days and tumor burden was quantified via bioluminescence (Average luminescence). Significant differences were determined using one-way ANOVA. (**h**) Images showing differences in ascites volume between eNK- and PBS-treated groups at the indicated times. Data are represented as means ± SD. *** $p < 0.001$; **** $p < 0.0001$.

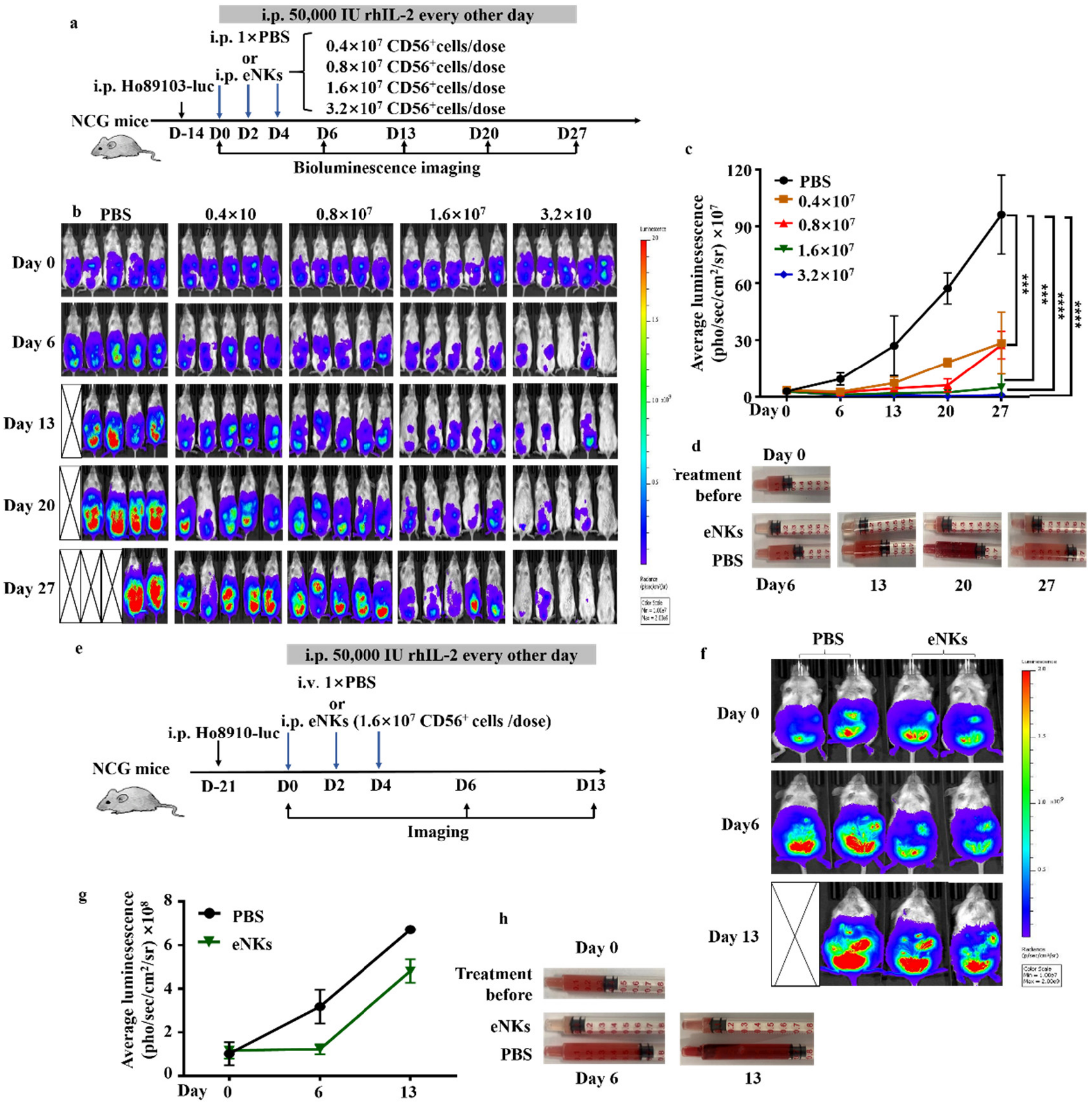

Supplement: Supplementary file 1 [file cancers-13-05866-s001.zip › cancers-1409133-SI.pdf]
